# Response of wheat, pea, and canola to micronutrient fertilization on five contrasting prairie soils

**DOI:** 10.1038/s41598-020-75911-y

**Published:** 2020-11-02

**Authors:** Noabur Rahman, Jeff Schoenau

**Affiliations:** Department of Soil Science, University of Saskatchewan, Saskatoon, SK Canada

**Keywords:** Plant sciences, Environmental sciences

## Abstract

A polyhouse study was conducted to evaluate the relative effectiveness of different micronutrient fertilizer formulation and application methods on wheat, pea and canola, as indicated by yield response and fate of micronutrients in contrasting mineral soils. The underlying factors controlling micronutrient bioavailability in a soil–plant system were examined using chemical and spectroscopic speciation techniques. Application of Cu significantly improved grain and straw biomass yields of wheat on two of the five soils (Ukalta and Sceptre), of which the Ukalta soil was critically Cu deficient according to soil extraction with DTPA. The deficiency problem was corrected by either soil or foliar application of Cu fertilizers. There were no significant yield responses of pea to Zn fertilization on any of the five soils. For canola, soil placement of boric acid was effective in correcting the deficiency problem in Whitefox soil, while foliar application was not. Soil extractable Cu, Zn, and B concentration in post-harvest soils were increased with soil placement of fertilizers, indicating that following crops in rotation could benefit from this application method. The chemical and XANES spectroscopic speciation indicates that carbonate associated is the dominant form of Cu and Zn in prairie soils, where chemisorption to carbonates is likely the major process that determines the fate of added Cu and Zn fertilizer.

## Introduction

Global food demand is likely to increase with growing population and rapid economic development, particularly in developing countries^1^. Increasing crop production to ensure food and nutritional security can have adverse impacts on agricultural land resources. A concern that has been brought forward is production of high yielding crop varieties without micronutrient fertilization, thus steadily depleting soil micronutrient content to below the critical level and triggering deficiency issues for further crop production^2^. Additionally, production of staple crops on micronutrient deficient soils results in not only yield reductions but also nutritional quality reductions associated with declining contents of important human nutrients like Zn and Fe^3^.

Improving micronutrient contents in regular diets is important in food and nutrition security, especially in South Asia and Sub-Saharan Africa where a majority of rural people are already vulnerable to micronutrient malnutrition problems^4^. While micronutrient enriched food products are needed for that region, agriculture in developed countries that export food products to the regions need to consider how their management practices are influencing the nutritional value of their food exports^5^. Therefore, an advanced agricultural strategy not only seeks to increase productivity in terms of yield but also the simultaneous enrichment of bioavailable micronutrients in staple food grains.

The combined efforts of crop development, including micronutrient efficient genotypes or biofortified crops and appropriate agronomic practices, have the potential to improve productivity and quality of agricultural products^3,4,6–8^. Among the agronomic approaches, fertilization is widely used to improve yield and mineral nutrient content of food crops. However, to adequately predict a crop response to micronutrient fertilization, knowledge of factors controlling micronutrient bioavailability in a soil–plant system is important^3,9^. Several of these important soil factors are soil pH, organic carbon and carbonate content, clay content, free lime, and amount and types of minerals that supply as well as fix micronutrients in the soil environment^3^. Such soil properties have direct influence on adsorption and precipitation of micronutrient elements to mineral and organic surfaces contributing to deficiency problems^10^. As well, an unwarranted application of micronutrient fertilizer may increase the risk of toxicity to a crop because of altered equilibria between soil solution and adsorption surfaces^3,10^. Understanding chemical behaviour of micronutrients under different soil-environmental conditions is therefore key to developing effective management practices.

Deficiencies in micronutrients such as Cu, Zn, and B deficiency are occasionally observed in soils of the Canadian prairies. However, in alleviating deficiencies, the soil test based recommendation is frequently noted to be inconsistent in terms of crop yield responses^11–13^. The reliability in predicting wheat yield response to Cu fertilization in suspected Cu-deficient mineral soils has been deemed unsatisfactory based on expectations produced through soil test assessments^11,12^. Similarly, recent work showed that Zn fertilization did not consistently improve the yield of lentil in different Saskatchewan soils, including those which were assumed to be Zn deficient and potentially responsive to Zn fertilization based on soil analysis^14,15^. Further, soil applied B fertilizer in soils with much lower extractable B (0.14 ppm) than the critical level failed to promote canola yield across the prairies^11,13^. This has led to the reliability of deficiency assessment methods (DTPA extraction of Cu and Zn; hot water extraction of B) to be questioned, and the critical level for achieving crop yield potential revised/lowered^11^. Debate has continued since, and the crucial role of site-specific soil properties in controlling efficiency of micronutrient fertilizer in modern multi-crop rotations (cereal-legume-oilseed) common on the Canadian prairies today requires re-evaluation^16^. In consideration of crop production and soil productivity sustainability, it is therefore important to explore nutrient management practices conducive to crop yield and quality improvement related to micronutrient management. The overall objective of the work is to evaluate the effect of different micronutrient fertilizer products and application methods on yield and micronutrient content of wheat, pea and canola, and their fate in five contrasting agricultural soils of the Canadian prairies.

## Materials and methods

### Experimental approach

The study was conducted during the crop growing season of 2015 in the polyhouse facilities of the University of Saskatchewan located on Preston Avenue in Saskatoon SK. The polyhouses were similar to greenhouses in that conditions were controlled, but were more similar to field conditions, using ambient light and temperature, but with water supply controlled and regulated. Evaluation of different micronutrient products and application methods for wheat (Cu), pea (Zn) and canola (B) crop growth and yield responses was conducted using five contrasting soils from the prairies. While the polyhouse cannot replicate the field conditions at each specific location from which a soil was taken, it provided an efficient mean of assessing and comparing crop responses to micronutrient fertilization in different soils under the same environmental conditions while also overcoming limitations associated with high variability across experimental field site areas. With environmental conditions (temperature, moisture, sunlight) standardized in the polyhouse, the influence of specific soil properties was more easily discerned.

### Soil, crop and fertilizer materials

Five different contrasting field soils with micronutrient fertility status ranging from marginal to deficient according to standard soil test method were used to grow wheat, pea and canola crops. These soils were collected at 0–15 cm depth from the field, air dried, homogenized and stored in plastic containers before use. The topsoil properties are suitable for crop establishment, and it retains majority of plant nutrients to promote crop growth. The five soils used are classified as (1) Whitewood association of Orthic Dark Grey Chernozem (Whitewood O.DGC, 52° 04′ 59.3″ N latitude, 102° 21′ 47.1″ W longitude); (2) Echo association of Brown Solodized Solonetz (Echo B.SS, 50° 47′ 27.4″ N latitude, 106° 30′ 49.0″ W longitude); (3) Whitefox association of Orthic Dark Grey Chernozem (Whitefox O.DGC; 53° 22ʹ 21.3ʺ N latitude, 103° 52ʹ 08.5ʺ W longitude); (4) Sceptre association of Orthic Vertisol (Sceptre O.V; 50° 45′ 1.5″ N latitude, 109° 17′ 55.9″ W longitude); and (5) Ukalta series of Orthic Black Chernozem (52° 22′ 38.8″ N latitude, 113° 05′ 10.7″ W longitude). Barley was grown as a forecrop in Ukalta series of Orthic Black Chernozem, while canola was the forecrop of other four soils.

Some important baseline physicochemical properties and initial nutrient concentrations were determined including texture, pH, electrical conductivity, organic carbon, and available micronutrient concentrations to characterize the soils and are provided in Table 1. Commercially available different micronutrient fertilizer forms were utilized. The product forms specifically used were sulfate salts and chelated fertilizer forms of Cu and Zn, and boric acid for B, respectively as these are commonly used fertilizer forms for Cu, Zn, and B fertilization on the prairies. The crop varieties used in this study include AC Waskada of hard red spring wheat (*Triticum aestivum*), CDC Meadow of yellow pea (*Pisum sativum*) and Liberty Link 150 of Argentine canola (*Brassica napus*).

**Table 1:** Basic soil properties and micronutrient fertility of experimental soils.

Soil	Basic properties^a^					Extractable micronutrient (mg kg^−1^)		
	pH	EC	FC	OC	Sand	Cu	Zn	B
Whitewood	6.9	0.16	29.6	2.98	49	0.83 (L)	1.44 (S)	0.95 (L)
Echo	6.5	0.16	18.9	2.01	45	0.73 (L)	1.18 (S)	1.55 (S)
Whitefox	5.0	0.53	35.5	2.21	57	1.31 (S)	1.79 (S)	0.54 (D)
Sceptre	7.6	0.49	42.4	1.70	7	1.86 (S)	0.87 (L)	1.15 (S)
Ukalta	5.5	0.6	37.6	2.55	81	0.38 (D)	2.47 (S)	0.95 (L)

^a^*EC *Electrical conductivity (mS/cm), *FC *moisture content at field capacity (%), *OC *organic carbon (%); Sand (%). Letter in parenthesis of extractable micronutrient represents: *D *deficient, *L *low, *S *sufficient.

### Experiment set-up, crop growth and management

The experiment was conducted using 2 L plastic pots (15 cm diameter and 15 cm depth). Each pot was filled with 1.8 kg of homogenized soil. Recommended rates of macronutrients for each crop were added to pots prior to seeding. Urea is added as external N source at the rate of 200 kg N ha^−1^ for wheat, 250 kg N ha^−1^ for canola, and 15 kg N ha^−1^ for pea, respectively. Lower nitrogen application for pea is considered to be compensated for by biological dinitrogen fixation, as commercial inoculant (*Rhizobium leguminosarum*) was applied to the pea seed. Other fertilizer blend applications are monoammonium phosphate (MAP) (11-52-0-0) and potassium sulfate (0-0-44-17) at the rate of 20 kg ha ^−1^ of P and S, respectively. Required amounts of fertilizer are calculated based on pot surface area and applied as broadcast followed by incorporation in soil.

For soil applied micronutrient treatments, the micronutrient salts were applied in solution form to facilitate uniform distribution in soil. The micronutrient solution was applied to the soil quantity for each pot and mixed thoroughly with the soil to simulate a broadcast and incorporation application. Application rates were selected based on typical micronutrient recommendation for crop production in Canadian prairies^12,13^. Rates of micronutrient applied represent the commonly recommended amounts when deficiency is detected (ALS Laboratories, Saskatoon, SK). Copper application rates were 5 kg Cu ha^−1^ of sulfate salts (CuSO_4_·5H_2_O = 20 kg ha^−1^) and 2 kg Cu ha^−1^ of chelated-Cu in soil-applied treatments, whereas Zn application rates in soils were 2 and 1 kg Zn ha^−1^ of ZnSO_4_·7H_2_O (11 kg ha^−1^) and chelated-Zn, respectively. Boric acid is applied in soil at the rate of 1 kg B ha^−1^. For foliar applications, the foliar application rate was 0.25 kg ha^−1^ for all of the micronutrient elements, and foliar application was made as chelated form of Cu and Zn as this is the main form in which the micronutrients are foliar applied (Saskatchewan Ministry of Agriculture Micronutrients in Crop Production Fact Sheet). Treatments are therefore (i) control; (ii) soil application (sulfate salt); (iii) soil application (chelated product) and (iv) foliar application (chelated product) of Cu and Zn for wheat and pea, respectively. For canola, the treatments of B are slightly modified as (i) control; (ii) soil application; (iii) foliar application (one time) and (iv) foliar application (two times) using boric acid. Two foliar application timings of B were used because canola response to B fertilization is sensitive to the crop stage at which the micronutrient is applied^17,18^. Fertilizer solutions for foliar application were prepared by dissolving each fertilizer product separately in 100 ml deionized water. To apply foliar treatments, a small plastic spray bottle was calibrated, and 1 ml of solution fertilizer was applied by pressing the spray trigger 5 times. Each treatment was replicated four times. After seeding, the pots were arranged randomly inside the polyhouse and position rotated at three-week intervals providing a completely randomized design.

Three plants were maintained in each pot with similar management practices including moisture content kept near field capacity by adding deionized water daily to desired weight. Soil temperature was monitored throughout the experimental period using HOBO temperature data loggers. The crops were seeded on June 10, 2015 and harvested at maturity at the end of August. The harvested plant samples were dried at 40 °C to a constant weight for grain and straw biomass yield measurements. All crop samples were threshed by hand and thoroughly ground using a tungsten grinder to prevent metal contamination. After crop harvesting, individual pot soils were homogenized by thorough mixing, air-dried at 30 °C, ground with a wooden rolling pin to break aggregates, sieved (< 2 mm fraction retained) and stored in plastic vials to await laboratory analysis.

### Chemical analysis for crop nutrition and soil fertility evaluation

Soil and plant samples were analyzed to determine plant uptake and distribution of applied fertilizer. Soil analyses conducted included physicochemical properties (pH, EC, organic and inorganic carbon content, texture, field capacity), measurements of extractable and total nutrient concentrations, and sequential fractionation to separate and identify general micronutrient species (Cu, Zn, and B) in the soil. The plant samples (grain and straw) were acid-digested to measure total uptake of nutrient elements. A Beckman 50 pH meter (Beckman Coulter, Fullerton, CA, USA) and an Accumet AP85 pH/EC meter (Accumet, Hudson, MA, USA) assemblage was used to measure soil pH and EC in 1:2 suspension (soil:water on a weight basis)^19,20^. The dry combustion method of carbon analysis^21^ was used to measure soil organic carbon (OC) using a LECO-C632 carbon analyzer (LECO Corporation, St. Josesph, MI, USA). The OC was measured following an HCl pre-treatment to remove carbonate content^22^. A modified pipette method was used to determine soil particle-size distribution^23^. The gravimetric method of determination of soil moisture was used to measure water content at field capacity in each of the soils^24^.

Soil available Cu and Zn were extracted with DTPA (0.005 M diethylene-triamine-penta acetic acid)^25^, and a hot water extraction method was used for available B^26^. For measuring total concentration of micronutrients, the USEPA 3051A method^27^ was used for sample digestion. Micronutrient distribution into various soil pools was determined using sequential chemical extraction method. The three-step modified BCR (Bureau of Reference) procedure was used for Cu and Zn^28^, while a five-step sequential extraction method was used for of B, respectively^26^. Concentrations of Cu and Zn were analyzed using a flame atomic absorption spectrophotometer (Varian Spectra 220 Atomic Absorption Spectrometer; Varian Inc., Palo Alto, CA, USA), while a 4100 MP-AES Microwave Plasma-Atomic Emission Spectrometer (Agilent Technologies) was used for B analysis.

## Statistical analysis

All data were subjected to analysis of variance (ANOVA) using PROC MIXED in SAS (Statistical Analysis System, Version 9.4 for Windows; SAS Institute, Cary, NC). The denominator degrees of freedom method (ddfm) option used the Satterthwaite method. Multi-treatment comparisons of variables were made using the Tukey’s studentized range test method of mean separation, while pdmix800 SAS macro^29^ was used to assign grouping. A probability level of P < 0.05 was chosen to establish statistical significance. Test of normality (PROC UNIVARIATE) and homogeneity of variances (Bartlett’s test) of all data sets were checked prior to the analysis.

### XANES data collection and analysis

The K-edge XANES spectra of Cu, Zn, and B were collected from samples of soil obtained at the end of the crop growth period using radiation facilities at Canadian Light Source (CLS), Saskatoon, Canada. The Hard X-ray Micro Analysis (HXMA) beamline (06ID-1), consisting a Si (III) double crystal monochromator and a 32 element Ge detector was used to scan soil samples in the energy range of 8950–9050 eV and 9600–9750 for Cu and Zn measurements, respectively. Prior to scanning samples, the beam energy was calibrated with a standard Cu or Zn foil to set the first inflection point of 8979 eV or 9569 eV. Spectra were collected in fluorescence mode on solid state samples at room temperature. Additionally, the Variable Line Spacing Planar Grating Monochromator (VLS PGM) beamline (11ID-2) was used for B spectra collection in the 180–220 eV region. The energy range of this beamline is 5.5–250 eV with a focus beam size of 500 µm × 500 µm. Boron spectra were simultaneously collected using both measurements modes of total electron yield (TEY) and total fluorescence yield (TFY). Multiple scans were collected to improve the signal to noise ratio. All XANES spectra were processed and analyzed by using Athena interface of Demeter 0.9.23 software^30^. With an extensive and detailed library of standard spectra developed by the soil chemistry lab at the University of Saskatchewan, the linear combination fitting (LCF) method was used for model development to estimate the proportion of the species in the samples.

## Results and discussion

### Efficacy of Cu, Zn, and B fertilization on increasing crop yield and nutrient utilization

#### Grain and straw yield

**Figure 1:**
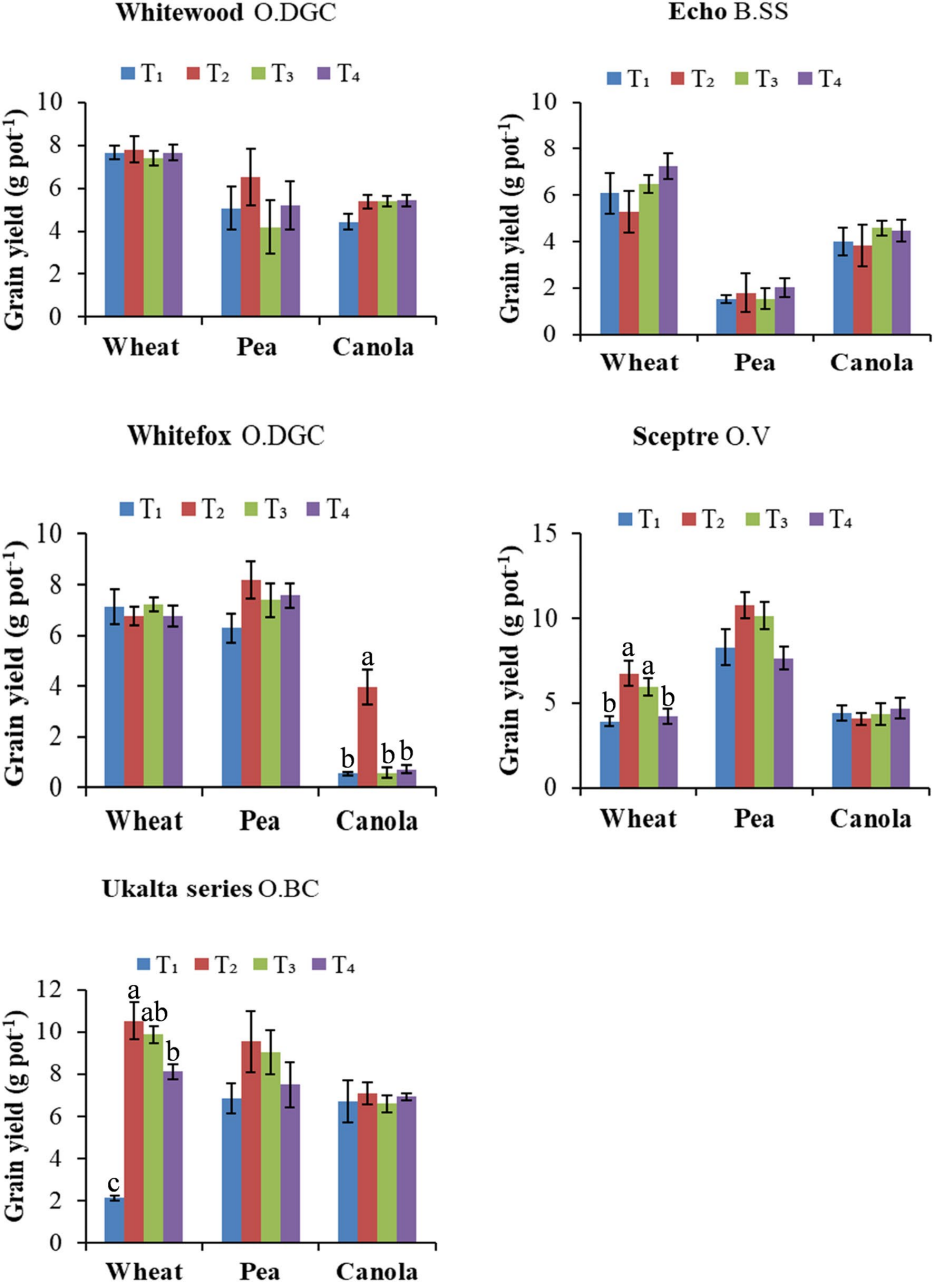
Grain yield responses of wheat, pea and canola to Cu, Zn, and B fertilization, respectively. Different micronutrient products and application methods are evaluated in five contrasting prairie soils. The soils are classified as (1) Whitewood O.DGC, (2) Echo B.SS, (3) Whitefox O.DGC, (4) Sceptre O.V, (5) Ukalta series, O.BC. Treatment evaluation includes T_1_: control; T_2_: soil application (sulfate salt); T_3_: soil application (chelated product) and T_4_: foliar application (chelated product) of Cu and Zn for wheat and pea, respectively. For canola, the treatments of B are tested as- T_1_: control; T_2_: soil application; T_3_: foliar application (one time) and T_4_: foliar application (two times) using boric acid. Treatment columns in each crop followed by the different letters are significantly different (p < 0.05). Error bar represents standard error of mean.

**Figure 2:**
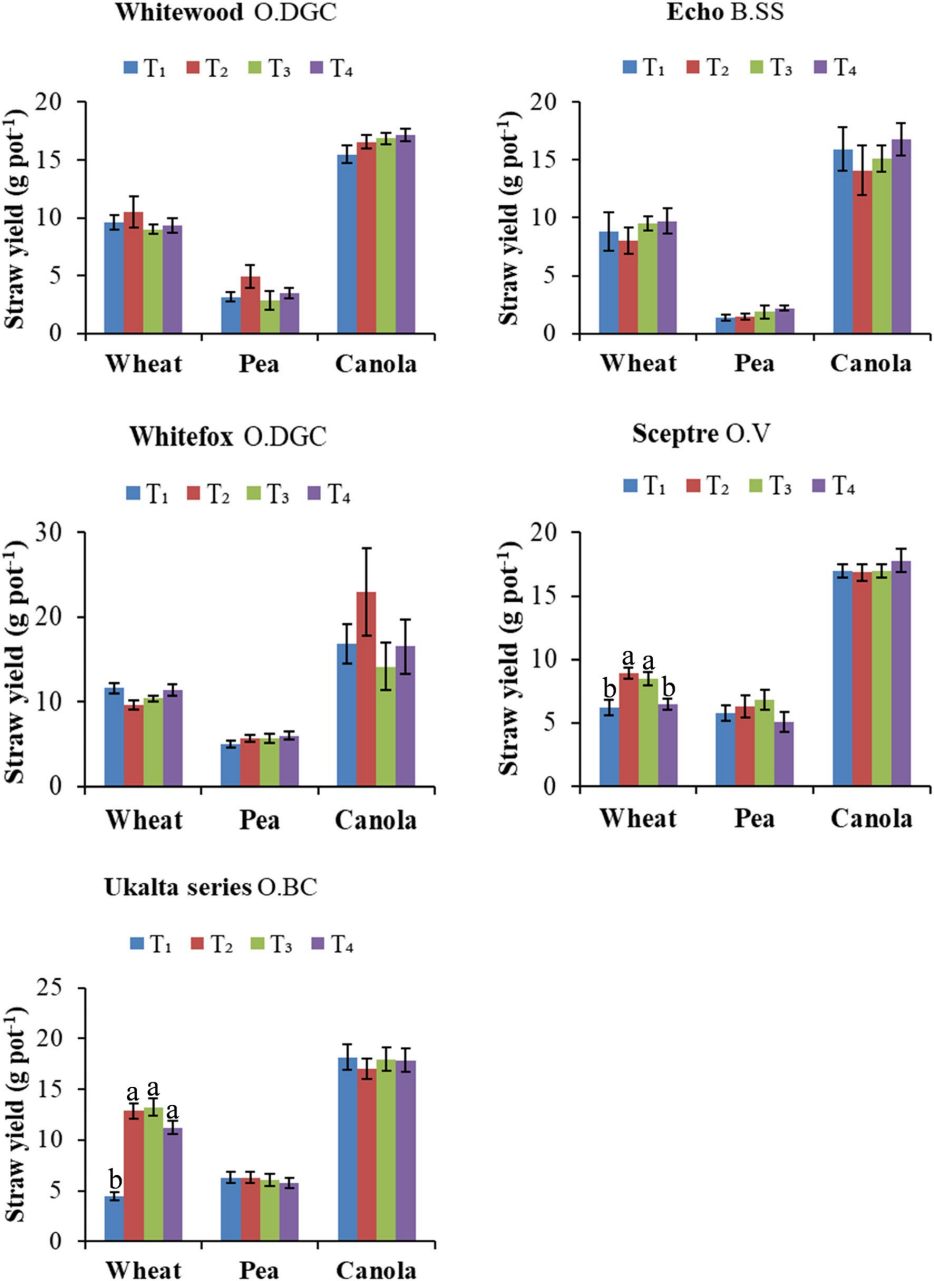
Straw yield responses of wheat, pea and canola to Cu, Zn, and B fertilization, respectively. Different micronutrient products and application methods are evaluated in five contrasting prairie soils. The soils are classified as (1) Whitewood O.DGC, (2) Echo B.SS, (3) Whitefox O.DGC, (4) Sceptre O.V, (5) Ukalta series, O.BC. Treatment evaluation includes T_1_: control; T_2_: soil application (sulfate salt); T_3_: soil application (chelated product) and T_4_: foliar application (chelated product) of Cu and Zn for wheat and pea, respectively. For canola, the treatments of B are tested as- T_1_: control; T_2_: soil application; T_3_: foliar application (one time) and T_4_: foliar application (two times) using boric acid. Treatment columns in each crop followed by the different letters are significantly different (p < 0.05). Error bar represents standard error of mean.

The grain and straw biomass yield of wheat were increased with Cu fertilization on two of the five soils (Ukalta and Sceptre) (Figs. 1, 2). Both soil and foliar application of Cu was effective in correcting deficiency problem in Ukalta soil, and this soil was critically deficient according to initial DTPA soil Cu level (0.4 mg kg^−1^ soil). The efficacy of soil applied Cu sulfate and Cu chelate was similar. However, the foliar fertilization produced significantly lower grain yield than the soil application of Cu. A similar pattern was observed for straw yield. This could be due to the lower application rates used in the foliar fertilization that may not be sufficient for yield optimization^31^. Compared to the control treatment, the increases in grain yield with Cu addition ranged from 281 to 394% in Ukalta soil. The Sceptre soil of high clay content was not Cu deficient according to DTPA extraction but did show a positive yield response to soil application, indicating that soil extraction and clay content are not always reliable indicators of response of cereal to Cu fertilization. This soil did have a very low supply rate (< 0.05 µg Cu 10 cm^−2^ 24 h^−1^) of available Cu according to assessment with resin membrane probe that may reflect limited mobility by diffusion in this high clay content soil. Various studies have shown the beneficial effect of Cu fertilization for optimizing wheat yields on Cu deficient mineral soils of Canadian prairies^12,31–34^. Previously, it was reported that soils containing DTPA-extractable Cu levels of less than 0.40 mg kg^−1^ are critically deficient, in which economic yield response to Cu fertilization is expected^12^. Although positive yield response can occur on marginally Cu deficient and sufficient soils with Cu fertilization^12,35^, economic benefits are less likely^12^.

Copper deficiency can alter plant metabolic and enzymatic activities, chlorophyll formation, and photosynthesis rates, with serious negative effect on crop productivity^36^. In wheat, the deficiency of Cu results in reduced vegetative growth, delayed maturity, and decreased grain and straw yields^37,38^. In Cu deficient Ukalta soil, the wheat plant showed typical Cu deficiency symptom including growing point death, tip withering, and delayed in heading under unfertilized control treatment. The grain yield reduction associated with Cu deficiency is primarily caused by pollen sterility that inhibits grain formation in cereal crops^39^. In this study, significant yield losses were observed due to poor grain formation. Further, the Cu deficiency can aggravate common diseases in cereals such as ergot, which is more prevalent in wheat and barley with Cu deficient soil conditions^35^. With ergot infection, the grains in wheat head are replaced by sclerotia, resulting in reduction in both yield and quality.

Pea yields were not significantly increased on any of the five soils by Zn fertilization. Initial DTPA extractable Zn content in four of these five soils was above the prescribed critical level of 0.5 mg kg^−1^^40,41^, and the other one was in the marginally deficient range. Usually, this type of fertility or deficiency is not likely to cause grain yield reduction in pulses as the plant can uptake sufficient amount of Zn for optimum crop growth. Similar study conducted with ten Saskatchewan soils found that Zn fertilization significantly increased the grain yield of lentil on a Zn deficient Echo and Ardill association soil^14^. However, fertilization with broadcast and incorporated Zn sulfate at two different field sites in Saskatchewan did not show economic yield responses in three different lentil classes^15^. The yield response to Zn fertilization typically occurs on sandy soils with low organic matter content by correcting critical deficiency problem. In Canadian prairie soils, there is some debate about what the critical deficiency levels of soil Zn are, as inconsistent crop yield responses to Zn fertilization are frequently noted^14,15,42^. Generally, DTPA extractable Zn of less than 0.5 mg kg^−1^ is considered as Zn deficient^40^, however, it is very likely to be influenced by environmental and soil conditions and certainly crop type. Most of the agricultural soils of prairies can supply adequate Zn for pulse production, and as such, the reported economic yield response to applied Zn is rare^14,15,42^. However, Zn fertilization could be considered as an effective agronomic approach for increasing grain Zn concentration in the edible parts of many crops^43^.

For canola, the Whitefox soil was the only soil in which grain yield of canola responded to application of B, and only soil application was effective. This soil had low soil extractable B (0.5 mg kg^−1^ soil), in the range where a response is considered possible or likely. Although canola is highly sensitive to B deficiency during the reproductive stage, the deficiency symptom also showed up at the vegetative stage. Surprisingly, foliar application treatments were ineffective in correcting the deficiency problem even with two applications at different stages. It was mainly due to low application rate which was not sufficient to correct the critical deficiency problem. There is some other evidence of yield improvement of canola by B fertilization in Canadian soils. For example, positive yield response to B addition was observed at a field site in northern Saskatchewan^44^, two B deficient sites in Quebec^45^, at a crop production center site in Manitoba, and in a controlled environmental study in Alberta^46^. Sometimes yield reduction with B fertilization is also observed in the field^13^. The variations in yield response of canola to B fertilization in soils deemed deficient in B according to soil test is more likely a consequence of many other factors affecting supply of available B to the plant, especially soil moisture status that could affect movement, root adsorption and translocation of B. Boron deficiency can also restrict canola root growth. In canola plants, B is more readily translocated through the xylem pathways by transpiration due to the lack of polyol-assisted phloem transport. However, the presence of bis-sucrose borate complex in phloem exudates indicates some B mobility through phloem^47^. Due to lack of ability to move from the leaves, the foliar fertilization at low rate during the vegetative growth stage of canola might be less effective especially with severe B deficiency and limited moisture soil conditions. A continuous supply of B is required for growth, pollination and seed development of canola^36,48^.

## Tissue concentration

Nutrient concentration in plant tissue can provide relevant information to verify a suspected deficiency. Increased tissue Zn concentration in pea was observed with Zn fertilization in Sceptre soil, while Cu and B concentrations were not responsive to fertilization in any of the studied soils (Table 2). The Cu concentration of 4.23–7.77 mg kg^−1^ in the grain and 2.19–4.56 mg kg^−1^ in the straw appears to be well above the critical levels^49^. Due to low recovery efficiency of applied Cu, the tissue concentrations were not influenced by fertilization. Critical whole plant Cu concentration of less than 2 mg kg^−1^^50^, 2.5 to 3.0 mg kg^−1^^51^ and 5 mg kg^−1^^49,52^ are reported as indicators of potential deficiency in wheat. However, the whole plant Cu concentration is often weakly correlated to grain and straw biomass yield responses to fertilization^34,50^. Copper fertilization of wheat with a tissue concentration of greater than 2 mg Cu kg^−1^ at mid-late tillering stages may not be effective for yield improvement^53^. Conversely, wheat yield response to Cu fertilization is expected with young leaf tissue concentration of less than 1.5 mg kg^−1^^54^.

**Table 2:** Cu, Zn, and B concentration in grain and straw of wheat, pea, and canola, respectively.

Treatment	Soil type									
	Whitewood		Echo		Whitefox		Sceptre		Ukalta	
	Grain	Straw	Grain	Straw	Grain	Straw	Grain	Straw	Grain	Straw
	**mg Cu kg**^**−1**^** in wheat**									
T_1_	5.19	3.21	5.09	2.19	6.03	3.95	7.22	4.16	4.23	2.90
T_2_	5.59	3.82	5.80	2.62	7.64	4.39	7.77	4.56	5.58	4.55
T_3_	5.40	3.56	5.80	2.55	7.14	4.23	7.66	4.46	4.49	3.84
T_4_	5.22	3.29	5.30	2.38	6.09	3.91	7.23	4.27	4.33	3.16
*p*-values	NS	NS	NS	NS	NS	NS	NS	NS	NS	NS
SEM	0.594	0.441	0.960	0.343	0.758	0.692	0.983	0.680	1.185	0.626
	**mg Zn kg**^**−1**^** in pea**									
T_1_	31.3	6.73	28.4	5.35	42.6	12.5b	16.7b	3.63b	33.6	5.54
T_2_	33.6	7.78	39.2	9.85	47.4	25.1a	19.0ab	4.46ab	40.3	10.7
T_3_	34.9	9.69	34.9	6.84	44.9	22.4a	23.0a	5.78a	36.0	7.83
T_4_	31.8	7.40	34.4	5.33	42.7	14.9b	20.4ab	4.46ab	34.7	5.64
*p*-values	NS	NS	NS	NS	NS	0.034	0.023	0.030	NS	NS
SEM	1.46	1.275	3.16	0.887	2.05	3.74	1.07	0.433	1.763	1.540
	**mg B kg**^**−1**^** in canola**									
T_1_	11.5	22.1	9.17	19.2	–^a^	17.6	12.6	21.7	9.9	20.9
T_2_	12.5	24.3	10.4	22.3	10.64	20.0	13.4	24.7	10.2	21.9
T_3_	11.5	22.2	9.28	20.6	–	18.8	12.7	23.0	10.0	21.0
T_4_	11.8	23.5	9.68	20.6	–	19.1	13.0	23.8	10.1	21.1
*p*-values	NS	NS	NS	NS	–	NS	NS	NS	NS	NS
SEM	0.396	0.619	0.285	0.796	–	0.948	0.471	1.18	0.147	0.412

Treatment evaluation includes T_1_: control; T_2_: soil application (sulfate salt); T_3_: soil application (chelated product) and T_4_: foliar application (chelated product) of Cu and Zn for wheat and pea, respectively. For canola, the treatments of B are tested as- T_1_: control; T_2_: soil application; T_3_: foliar application (one time) and T_4_: foliar application (two times) using boric acid.

*NS *Not significant (p > 0.05), *SEM *Standard error of mean (n = 4).

^a^Boron concentration in canola grain of Whitefox soil were not measured due to insufficient amount of grain. Treatment columns of grain and straw of each crop followed by the different letters are significantly different.

The critical Zn concentration in young leaf tissues of wheat is reported as less than 14 mg kg^−1^^55^, while for cowpea it ranges from 13 to 50 mg kg^−1^ in different parts of leaves^56^. Considerable variation in critical Zn concentration were observed in various pulse crops and were also affected by growth period^57^. For field pea, the critical Zn concentration of grain at maturity is less than 20 mg kg^−1^^57^. We found grain Zn concentration of 16.7 mg kg^−1^ in the unfertilized treatment (control) of Sceptre soil, which indicates that pea production might have been suffered from Zn deficiency and there was a trend for grain yield to be higher in this soil with Zn fertilization. Moreover, increasing tissue Zn concentration with fertilizer application indicates that Zn fertilization is an effective agronomic approach to overcome deficiency problem and to achieve Zn biofortification in pea. An overall increase of 20% in the lentil grain Zn concentration was observed with Zn application on a Brown Chernozem soil from Saskatchewan^14^.

The soil placement of B tended to increase B concentration in seed and straw of canola, but the effect was not significant at P < 0.05 (Table 2). The mean straw B concentration at maturity in the control treatment of Whitefox soil was 17.6 mg kg^−1^, and the canola in this soil showed B deficiency symptoms at vegetative growth stages and increased grain yield with soil application of B. Our results showed that there was an increasing trend of tissue B concentration in canola with soil fertilization. The critical deficiency concentration of B in mature crop plants ranges from 5 to 39 mg kg^−1^^58^ and greatly reflects differences in the B requirement of different crop species. Boron concentration of canola leaves was lower than 15 mg B kg^−1^ when it was grown on B deficient soils^45^. The optimum tissue concentration of B at flowering of canola is reported as 29 mg B kg^−1^^46^, and B fertilization may not be required if the tissue concentration is within the range of 20–30 mg B kg^−1^^44^.

### Cu, Zn, and B in post-harvest soil

#### Soil extractable Cu, Zn, and B

Unused available nutrients remaining in post-harvest soil from pre-seeding fertilizer application, termed residual or “legacy” fertilizer nutrient, can potentially benefit future crop nutrition. Usually, the bioavailability of soil applied micronutrients varies considerably with soil type as their concentration in solution is greatly influenced by soil adsorption and desorption reactions they undergo over time as well as removal through root assimilation^59^. In this study, soil application of sulfate salt and chelated forms of Cu increased the concentration of post-harvest DTPA soil extractable Cu in all five soils (Table 3). Increased post-harvest extractable Zn concentrations were also observed with soil placed Zn sulfate fertilizer, while three of five soils also showed increased residual available Zn with chelated Zn fertilizer application to soil. The soil residual B was also significantly increased with soil applied boric acid fertilizer. These observations indicate that critical deficiency problem can be corrected following crops in rotation can be benefited from soil application of micronutrient fertilizer. In agreement with these findings, there was a significant increase in hot water-soluble B in post-harvest soils of a rice-vegetable based cropping system experiment conducted recently in India^60^. A Cu deficiency problem in a Black Chernozemic soil from central Alberta was effectively corrected by soil application of either sulfate salt or chelated Cu fertilizers, and a residual benefit of increased wheat yield was obtained after four years of chelated Cu addition^32^. Our results agree well with a field experiment conducted in Pakistan, which reported that post-harvest DTPA extractable Cu and Zn levels were increased with soil application of sulfate fertilizers^61^. Further, the observed differences in residual Cu and Zn between sulfate and chelated forms is largely explained on the basis of application rate. Zinc fertilization with increased rates did show a significant increase of DTPA-available Zn in a pot study conducted in India^62^. In Saskatchewan, the DTPA-extractable soil Zn level was increased with soil application of ZnSO_4_ at 10 kg Zn ha^−1^^42^. Additionally, residual benefits with application of chelated forms were observed for a longer period than sulfate formulation of Zn fertilizer^63^.

**Table 3:** Post-harvest soil extractable Cu, Zn, and B in five contrasting soils used to grow wheat, pea, and canola, respectively.

Treatment	Soil type				
	Whitewood	Echo	Whitefox	Sceptre	Ukalta
	**mg Cu kg**^**−1**^** soil**				
T_1_	0.86c	0.70b	1.31c	1.83c	0.38b
T_2_	3.07a	1.79a	4.56a	3.80a	1.77a
T_3_	1.98b	1.35a	2.34b	3.47ab	0.60ab
T_4_	1.10c	0.74b	1.45bc	1.95bc	0.30b
*p*-values	< 0.0001	0.0008	< 0.0001	0.009	0.013
SEM	0.206	0.124	0.188	0.316	0.275
	**mg Zn kg**^**−1**^** soil**				
T_1_	1.44c	1.18b	1.73c	0.79b	2.43b
T_2_	2.43a	1.89a	4.66a	2.03a	3.58a
T_3_	1.86b	1.54a	3.01b	1.20b	2.60b
T_4_	1.42c	1.15b	1.67c	0.92b	2.29b
*p*-values	< 0.0001	0.0002	< 0.0001	0.0005	0.0002
SEM	0.107	0.072	0.151	0.107	0.129
	**mg B kg**^**−1**^** soil**				
T_1_	1.09b	1.63b	0.51	1.22b	0.96b
T_2_	1.50a	2.48a	0.72	2.04a	1.92a
T_3_	0.85b	1.73b	0.56	1.27b	0.84b
T_4_	0.96b	1.83b	0.50	1.33b	0.95b
*p*-values	0.0003	0.006	NS	< 0.0001	< 0.0001
SEM	0.057	0.129	0.06	0.589	0.073

Treatment evaluation includes T_1_: control; T_2_: soil application (sulfate salt); T_3_: soil application (chelated product) and T_4_: foliar application (chelated product) of Cu and Zn for wheat and pea, respectively. For canola, the treatments of B are tested as- T_1_: control; T_2_: soil application; T_3_: foliar application (one time) and T_4_: foliar application (two times) using boric acid. Means in treatment column followed by the different letters are significantly different.

*NS *Not significant (p > 0.05), *SEM *Standard error of mean (n = 4).

### Chemical speciation

Chemical partitioning or speciation through sequential fractionation schemes is used to determine the amounts and distribution of micronutrient among different chemically extracted forms. It has proven useful in providing a better understanding of mobility and bioavailability behavior in soils. The total amounts and relative chemical forms of Cu, Zn, and B sequentially extracted from post-harvest soils used in the current study are shown in Table 4. Total concentration of micronutrients in all soils were elevated with soil fertilization, and significant redistribution to labile forms was also observed. The majority of the soil applied Cu and Zn were retained in the soil solution-carbonate-exchangeable (F_1_) and oxyhydroxide fractions (F_2_). The adsorption of Cu and Zn to carbonates, (oxy)hydroxide minerals and organic matter are recognized as the main mechanism of retention of metal micronutrients in soils^64,65^. Added Cu and Zn are likely to be occluded in soil as carbonate salts through chemisorption process. Increased Cu concentration in the organic bound fraction was also observed with Cu-sulfate fertilizer addition in Whitefox, Sceptre, and Ukalta soils. The entry of Cu sulfate into the organic bound fraction did not appear to be related to organic carbon content of these soils. However, Cu is known to have strong adsorption affinity to organic matter^7,36^, reducing its availability over a long period of time after fertilization. Conversely, Cu tends to form chelated complexes with dissolved organic matter, which increases the potential for plant uptake^66^. Our results also indicate that the largest fraction of Cu and Zn in the experimental soils is associated with the residual fraction, which is not readily available for crop utilization and of which the majority is likely Cu locked in recalcitrant primary and secondary minerals. Soil applied B was mostly detected in hot water soluble and specifically adsorbed fractions. Our results indicate that B deficiency problem can be corrected effectively by soil fertilization. Similar increase of B concentration in readily soluble and specifically bound fractions was reported in a field study conducted in India^60^. We also observed that the major portion of total soil B was retained in residual form. Another B fractionation study using Brown and Grey Luvisol soils from Saskatchewan found similar results^26^. The residence of much the applied micronutrient in labile forms after harvest of the first crop supports potential residual benefit to following crops.

**Table 4:** Chemical fractionation of Cu, Zn, and B in five contrasting soils as influenced by different products and application methods of micronutrient fertilization.

Soil	Treatment	mg Cu kg^−1^ soil					mg Zn kg^−1^ soil					mg B kg^−1^ soil				
		F_1_	F_2_	F_3_	F_4_	F_5_	F_1_	F_2_	F_3_	F_4_	F_5_	F_1_	F_2_	F_3_	F_4_	F_5_
Whitewood	T_1_	0.48c	0.63b	3.08	6.88	11.1b	0.92c	5.02c	8.50	32.1	45.6	0.34b	0.15b	1.75	77.6	80.9
	T_2_	1.90a	1.08a	3.97	6.51	13.5a	1.84a	6.20a	8.41	33.4	49.8	0.56a	0.26a	1.91	77.4	81.6
	T_3_	1.51b	0.86ab	3.68	6.42	12.5ab	1.25b	5.87b	8.48	33.9	49.5	0.36b	0.19b	1.94	77.5	80.9
	T_4_	0.45c	0.59b	3.13	7.07	11.2b	0.91c	5.02c	7.95	33.0	46.8	0.40b	0.16b	1.64	77.7	80.9
*p*-values		< 0.0001	0.037	NS	NS	0.037	< 0.0001	< 0.0001	NS	NS	NS	0.006	0.009	NS	–	–
SEM		0.086	0.115	0.293	0.202	0.565	0.081	0.095	NS	1.59	1.68	0.039	0.020	0.117	–	–
Echo	T_1_	0.38b	0.56b	2.03	5.18	8.15b	0.77bc	4.24b	6.27	33.9	45.2	0.54b	0.34	2.58	71.7	76.8
	T_2_	1.17a	1.14a	2.58	5.15	10.1a	1.11a	5.04a	6.75	34.4	47.3	0.99a	0.41	2.56	70.9	77.4
	T_3_	0.93b	1.20a	2.77	6.17	10.8a	0.90b	4.33b	6.92	35.8	47.9	0.46b	0.29	2.61	71.7	76.8
	T_4_	0.36c	0.48b	2.13	5.32	8.30b	0.71c	4.13b	6.46	34.3	45.6	0.52b	0.29	2.70	71.5	76.8
*p*-values		< 0.0001	< 0.0001	NS	NS	0.0002	0.003	0.002	NS	NS	NS	0.002	NS	NS	–	–
SEM		0.077	0.077	0.167	0.312	0.336	0.061	0.135	0.155	1.36	1.39	0.082	0.049	0.459	–	–
Whitefox	T_1_	0.61c	0.58c	1.65b	4.65	7.49c	1.44c	3.46	8.31	18.0	31.2	0.35	0.11	2.48	51.8	55.2
	T_2_	2.30a	2.05a	3.30a	4.50	12.2a	4.08a	3.96	8.17	18.3	34.5	0.58	0.13	2.37	51.9	55.7
	T_3_	1.23b	1.13b	1.91b	5.27	9.64b	3.51b	3.83	8.34	18.4	34.1	0.38	0.09	2.31	51.9	55.2
	T_4_	0.60c	0.65c	1.72b	4.23	7.19c	1.46c	3.48	8.08	19.5	32.5	0.45	0.10	2.40	51.8	55.2
*p*-values		< 0.0001	< 0.0001	< 0.0001	NS	< 0.0001	< 0.0001	NS	NS	NS	NS	NS	NS	NS	–	–
SEM		0.156	0.504	0.148	0.250	0.224	0.156	0.145	0.218	1.46	1.37	0.095	0.037	0.072	–	–
Sceptre	T_1_	0.68b	3.04b	3.88b	11.8	19.4b	0.41b	6.85c	12.3	61.3	80.9b	2.40b	1.43	4.35	144	153
	T_2_	1.78a	4.29a	4.78a	11.4	22.3a	1.31a	8.68a	12.9	61.6	84.5a	3.05a	1.46	4.57	143	154
	T_3_	1.47a	4.15a	3.72b	11.4	21.7a	1.04a	7.52b	12.8	63.0	84.3a	2.32b	1.15	4.17	144	153
	T_4_	0.67b	2.90b	3.68b	12.4	19.7b	0.39b	7.19bc	12.2	59.8	79.6b	2.13b	1.14	4.52	144	153
*p*-values		0.0001	0.001	0.002	NS	0.011	< 0.0001	< 0.0001	NS	NS	0.009	< 0.0001	NS	NS	–	–
SEM		0.139	0.228	0.164	0.467	0.597	0.109	0.162	0.256	0.906	0.994	0.093	0.104	0.113	–	–
Ukalta	T_1_	0.31b	0.09c	1.57b	4.29	6.26b	1.95b	4.26b	8.07	19.8	34.1	0.44b	0.79	2.63	47.0	51.8
	T_2_	1.27a	0.69a	3.10a	2.80	7.86a	2.88a	5.53a	9.79	20.0	38.2	0.87a	0.95	2.79	45.7	52.2
	T_3_	0.72a	0.39b	2.34ab	4.00	7.45a	2.15b	5.10a	7.90	19.2	34.3	0.41b	0.87	2.72	47.0	51.8
	T_4_	0.28b	0.11c	1.68b	3.83	5.91b	1.96b	4.06b	7.91	20.1	34.0	0.47b	0.81	2.77	46.8	51.8
*p*-values		< 0.0001	< 0.0001	0.003	NS	< 0.0001	0.015	< 0.0001	NS	NS	NS	0.023	NS	NS	–	–
SEM		0.075	0.028	0.248	0.270	0.216	0.193	0.162	0.673	1.83	1.82	0.100	0.102	0.116	–	–

Different fractions are F_1_: soil solution-carbonate-exchangeable fraction (Cu and Zn) or specifically adsorbed fraction (B); F_2_: oxyhydroxide fraction; F_3_: organic-bound fraction; F_4_: residual fraction; F_5_: total concentration in soil. The total concentration of B was measured in composite sample, and statistical analyses were not performed for residual fraction and total boron. For a soil, treatment columns followed by the different letters are significantly different.

*NS *Not significant (p > 0.05), *SEM *Standard error of mean (n = 4).

Understanding the effect of soil properties on micronutrient bioavailability is of great importance for developing an effective nutrient management plan, which will help to optimize crop growth and productivity response to micronutrient fertilization. The bioavailability of Cu and Zn is highly dependent on soil solution concentration and speciation of the elements, because the free cations can easily undergo surface adsorption reactions with various soil components^7,36^. Usually, Cu and Zn adsorptions to the natural oxyhydroxide and carbonates largely controls mobility and bioavailability in agricultural system. In soils, the adsorption processes are classified as specific and non-specific reactions^65^. The specific adsorption of micronutrients typically occurs with soil organic and inorganic colloids which includes organic matter, clay minerals, Fe and Mn (hydro)oxides, and carbonates^67^. Moreover, the specific adsorption of micronutrients is influenced by soil pH and redox reactions through its effects on surface charge characteristics^7^. For example, with increasing soil solution pH, the surface negative charge of adsorbent increases through deprotonation process which in turn facilitates increased adsorption of micronutrient cations. In general, micronutrient metal bioavailability decreases as soil pH increases.

### Spectroscopic speciation

**Figure 3:**
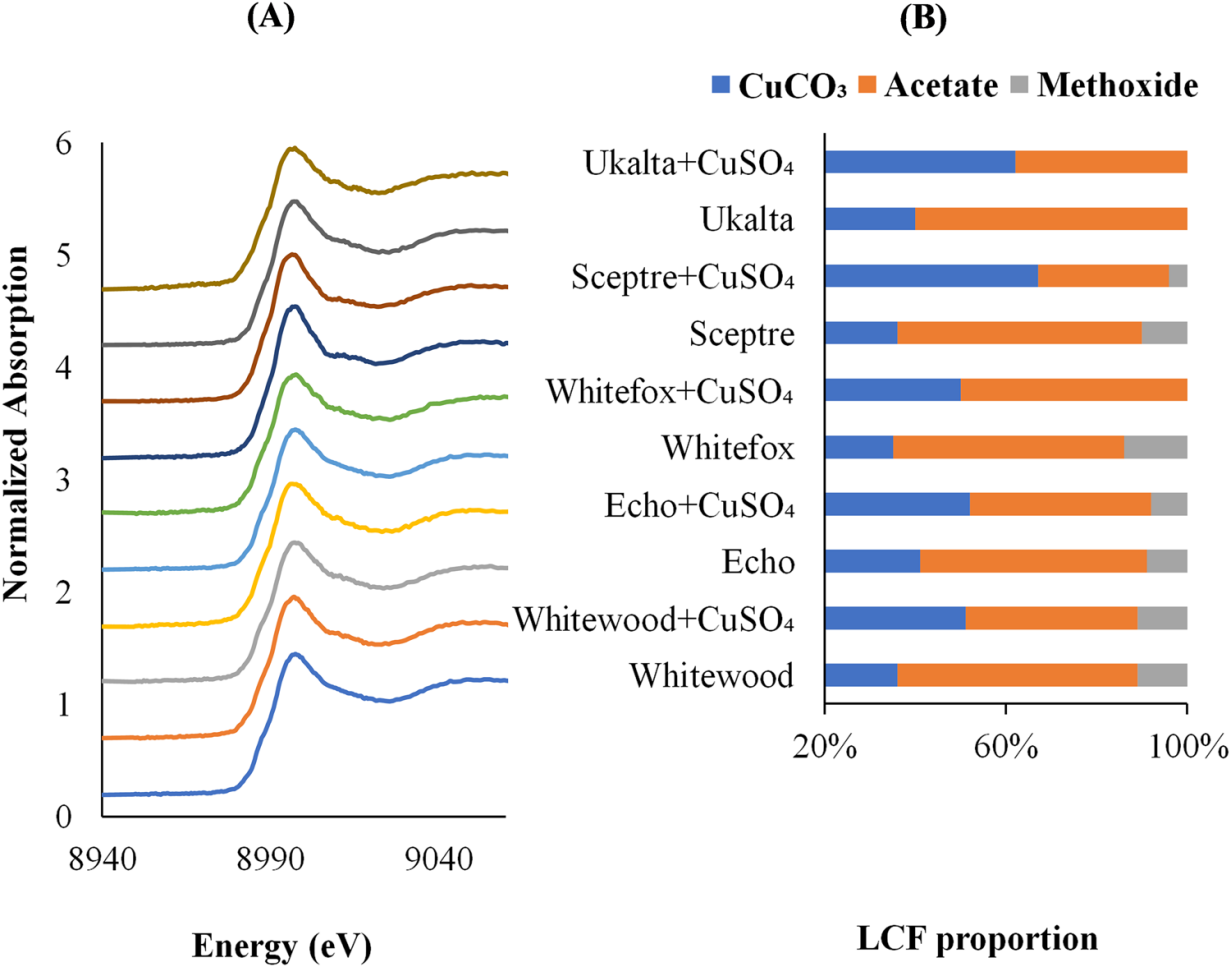
(**A**) Normalized Cu XANES K-edge spectra of five contrasting agricultural soils of the Canadian prairies without and with CuSO_4_ fertilizer amendment. (**B**) Results of linear combination fit, showing the relative proportions and differences in Cu speciation among soils and treatments.

**Figure 4:**
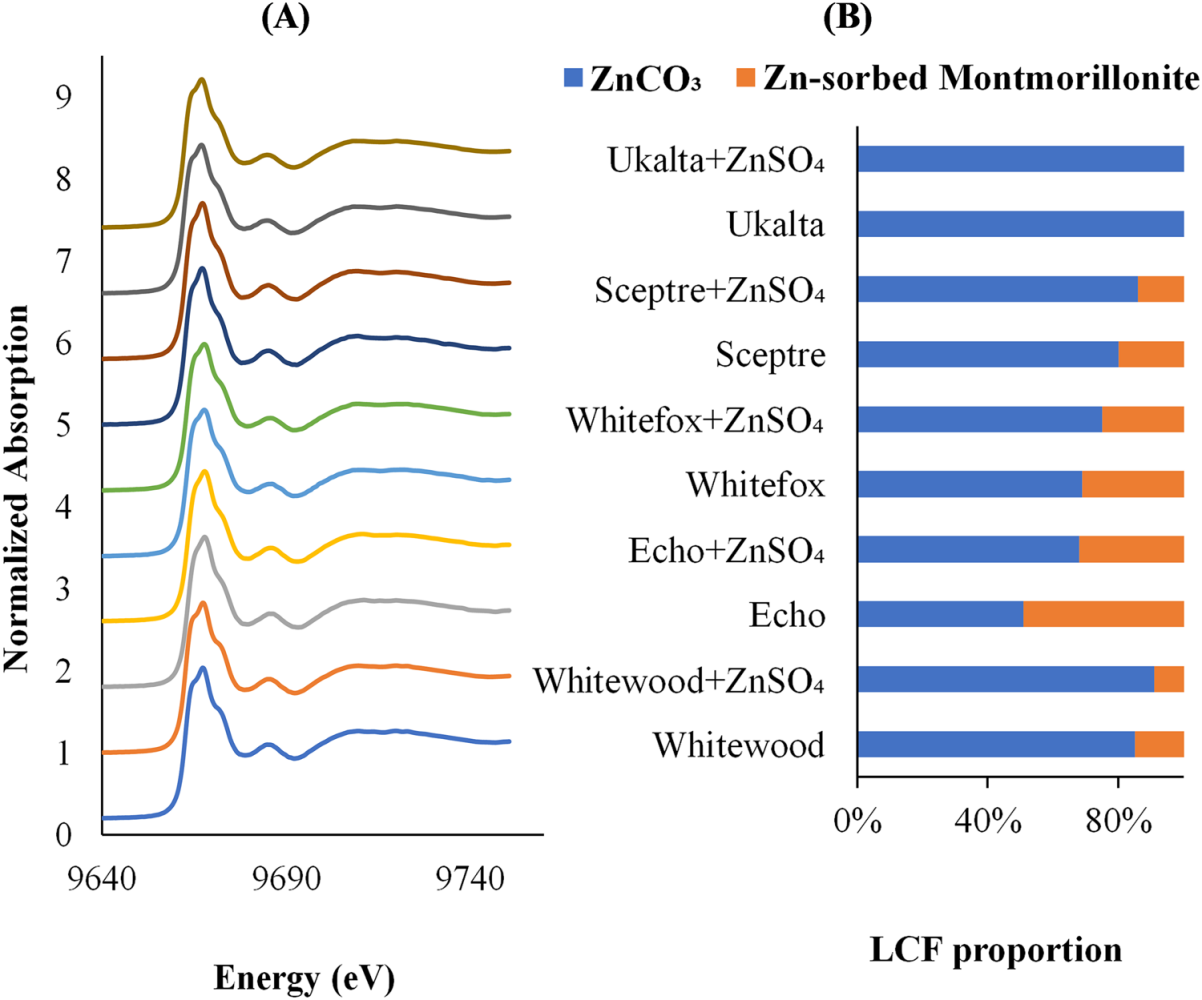
(**A**) Normalized Zn XANES K-edge spectra of five contrasting agricultural soils of the Canadian prairies without and with ZnSO_4_ fertilizer amendment. (**B**) Results of linear combination fit, showing the relative proportions and differences in Zn speciation among soils and treatments.

Bulk Cu and Zn K-edge XANES spectra of the five contrasting soils, with and without amendment of fertilizer, are presented in Figs. 3 and 4. Results of linear combination fittings of XANES spectra showed that the dominant Cu species identified were Cu carbonate, Cu acetate, and Cu methoxide in the experimental soils. However, Cu methoxide was not identified in coarse textured Ukalta soil. For Zn, the predominant species were carbonate and Zn-orbed montmorillonite (Fig. 4). Previous research has indicated that association with carbonate minerals and organic matter are major sorption processes of Cu and Zn in soils^68–70^. The qualitative analysis of B K-edge XANES spectra helped to identify trigonal BO_3_ species as diluted samples are not providing accurate B speciation in agricultural soils.

X-Ray Absorption Fine Structure (XAFS) spectroscopy is a valuable tool to probe the metal complexes at the surfaces of mineral oxides, silicate clays and soil organic matter. Using EXAFS, earlier study revealed that Cu^2+^ and Zn^2+^ forms mononuclear innersphere complexes at the calcite surface through substitution of these metals in the Ca site^71^. Previous study found similar local coordination of Cu^2+^ at the calcite surface in presence of dissolved humic acid^66^. However, the adsorption of Cu^2+^ to calcite surfaces was decreased with increased concentration of humic acid^66^. Further, the adsorption sites of inorganic minerals might be blocked with humic acid coating, and it may result in low adsorption affinity for metal micronutrients^72^. The molecular structure of Cu adsorbed on several types of natural organic materials indicate that Cu was forming a five-member chelating ring with amine, carboxyl, and carbonyl functional groups of organic compounds^73^. Further, earlier study used XANES and EXAFS spectroscopy to examine Cu speciation in contaminated soils and indicated that Cu was predominantly adsorbed on soil organic matter, rather than carbonates minerals or Fe oxides^74^. Similar Cu speciation results were found for calcareous agricultural soils^75,76^.

By use of XAFS spectroscopy, the majority of Zn sorption complexes have been identified at the surface of clay minerals. At montmorillonite surface, Zn forms outer sphere complexes with an octahedral coordination^77^. On contrary, Zn adsorption complexes were in tetrahedral coordination and forming inner-sphere complexes at the calcite surface^71^. Previous study also reported that Zn sorption onto montmorillonite surface was initially faster presumably with higher reactive surface areas where 40% of Zn was taken up within the first 20 min, while 6 months was needed to adsorb 80% of Zn^77^. Apart from outer- and inner-sphere complexation, Zn is more likely to be incorporated into surface precipitate phases such as a mixed Zn-Al layered double hydroxide (Zn-Al LDH) or Zn phyllosilicate phase^78^. However, Zn precipitate formation usually occurs with increased surface loadings, which is less likely in agricultural system.

Advanced spectroscopy like B K-edge XANES study of B coordination indicates that both trigonal and/or tetrahedral inner-sphere complexes are formed on soil mineral and organic surfaces^79^. Similar to Cu and Zn, the surface adsorption reaction and complexation mechanisms of B are governed by many soil factors including pH, organic matter and clay mineral types^79–81^. For example, within the pH range of 5.0 to 7.0 trigonal species B(OH)_3_ predominately occurs in soil while tetrahedral species B(OH)_4_ is observed with increased pH (> 7 or alkaline soil condition)^82^.

## Conclusion

The results of this polyhouse study with five contrasting agricultural soils of the prairie region indicate that Cu and B fertilization was effective in correcting deficiency limitation for growth of wheat and canola, respectively. The Zn deficiency appeared to be of less concern than Cu and B deficiency as a micronutrient limiting crop production in the prairie soils. Increased concentrations of extractable Cu, Zn, and B were observed post-harvest from soil-applied fertilizers, which may favor performance of following crops grown in rotation. The chemical and spectroscopic speciation results indicate that carbonate associated is the dominant form of Cu and Zn, and carbonate adsorption is likely the major process determining the fate of added fertilizer product.
